# Supplementary figures and images for: Y-maze performance predicts refined motor learning in mice
Source: PLoS One. 2026 Jul 1;21(7):e0352676. doi: 10.1371/journal.pone.0352676 (PMC13322532; doi:10.1371/journal.pone.0352676)

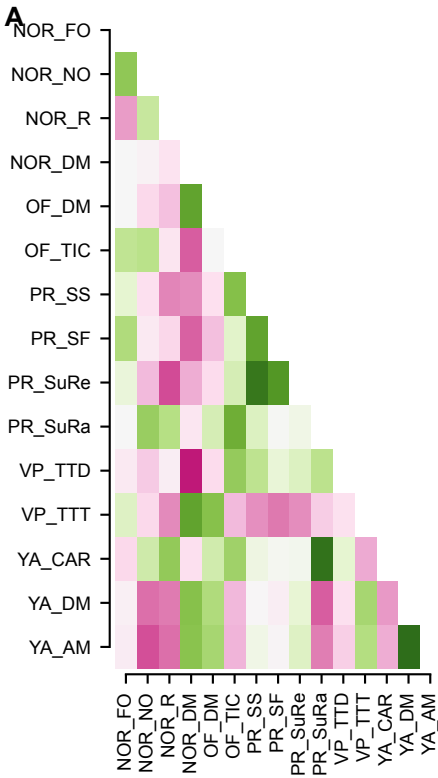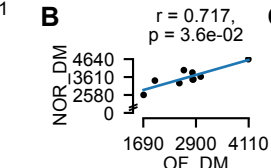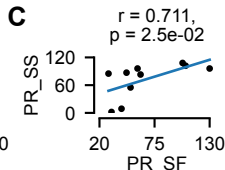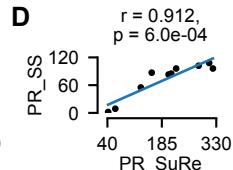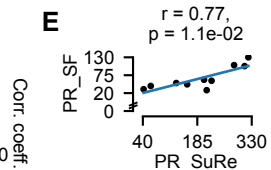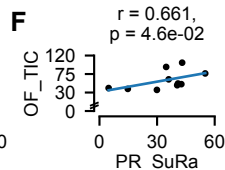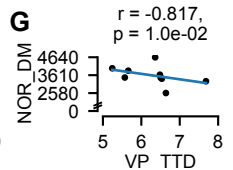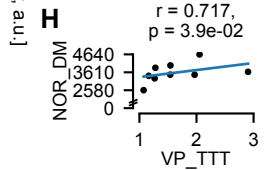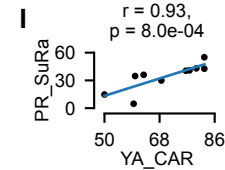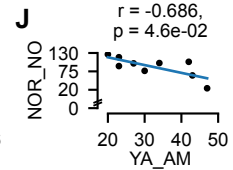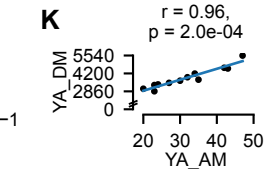

Supplement: S1 Fig — Abbreviations: NOR (Novel Object Recognition); OF (Open Field); PR (Pellet Reaching); VP (Vertical Pole); YA (Y-maze); FO (Familiar Object); NO (Novel Object); R (Ratio); DM (Distance Moved); TIC (Time In Center); SS (Sum of Success); SF (Sum of Fail); SuRe (Sum of all Reaches); SuRa (Success Ratio); TTD (Time To Descend); TTT (Time To Turn); AM (Alternations Maximum – total number of possible alternations); CAR (Correct Alternation Ratio). (PDF) [file pone.0352676.s001.pdf]
