# Supplementary material for: Y-maze performance predicts refined motor learning in mice
Source: PLoS One. 2026 Jul 1;21(7):e0352676. doi: 10.1371/journal.pone.0352676 (PMC13322532; doi:10.1371/journal.pone.0352676)
Supplement: Attachment — Submitted filename: 20260525-CiralliEtAl-Reviews_PLOSOne.pdf [file pone.0352676.s003.pdf]

Reviewers' comments

We are thankful for the insightful feedback. Comments with punctual suggestions were addressed directly in the revised manuscript, while concerns requiring explanations were answered below. All line numbers refer to the revised version.

Reviewer #1

To strengthen the rigor and impact of your findings, I have several critical points that require clarification and further consideration.

1. On the Core Conclusion and the Claim of "Prediction"

The central conclusion posits that the Y-maze and Vertical Pole tasks are "suitable predictors" of pellet-reaching performance. However, the study design is inherently cross-sectional and correlational, not longitudinal or predictive. All tasks were assessed within a similar timeframe. Can you provide stronger justification or refine the language to clarify how a correlation measured concurrently translates into a reliable prediction of future performance on a distinct, more complex task? Is the implied predictive power based on shared neural substrates (e.g., hippocampal-striatal circuits) or a more general "learning readiness" trait? The term "predictor" may be too strong without evidence from a true pre-screening followed by a later assessment of motor learning.

We understand that the previous version of our study only applied correlation analyses. Therefore, we now included a full predictive validity analysis using partial correlations, multiple linear regression, leave-one-out cross-validation (LOOCV), and bootstrap confidence intervals. Our new findings (Figure 4) further support the conclusion that Y-maze performance predicts pellet-reaching performance.

2. On Experimental Design and Confounding Factors

Fixed Order of Testing: The Methods indicate that all behavioral assays (Open Field, NOR, Y-maze, Vertical Pole) were conducted prior to the Pellet Reaching task. This fixed order is a significant confound. Could the testing sequence itself introduce practice effects, fatigue, or cumulative stress that systematically influences performance in the final, most complex task? For instance, are mice that are more active/less anxious in the initial tasks also the ones that show higher engagement in the final motor learning task? While you briefly acknowledge this in the discussion (lines 378-386), a deeper analysis is needed to disentangle whether the observed correlations reflect intrinsic ability links or are artifacts of the testing schedule.

All behavioral tasks used in our study were brief, lasting no more than 10 minutes, and took place over different days, and our testing protocol relies on the animal's natural innate rhythm, as it is not subjected to forced exhaustion methods such as forced swim test or fixed speeds in treadmills, therefore it is highly unlikely that the testing sequence could cumulatively introduce fatigue or stress. As indicated in our experimental timeline (Figure 1A), the open field and the NOR were conducted on consecutive days, then the Y-maze and vertical pole tests were performed 3 days after the previous test, and the pellet-reaching was performed one week after the vertical pole. In addition, we showed in Figure 2A-B that although mice moved less and slower in the NOR test compared to the open field (comparing the first 5 minutes of the NOR vs the open field to ensure length consistency as explained in the results section), they still

covered high distances at a reasonable speed, indicating that this decrease could be due to exploration of the objects. Also there were no differences between distance moved and speed in open field compared to Y-maze subsequent test, which indicates no fatigue between those tests. Further, we found no correlation between the distance moved in the open field and any pellet-reaching measurement ($r < 0.4$, $p > 0.223$). We agree that discussing these points would be beneficial, and added a paragraph to the discussion section highlighting this concern (lines 348-357):

"To reduce the risk of fatigue and stress affecting performance, all behavioral tests were limited to a maximum of 10 minutes per day. This approach minimized the likelihood that fatigue would significantly influence task outcomes. Additionally, our testing protocol relied on the animal's natural innate rhythm, as it is not subjected to forced exhaustion methods such as forced swim test or fixed speeds in treadmills. This allows the animal to control its own performance in accordance with its individual pace and well-being, which may contribute to a reduction in stress levels. Also, we also observed that although mice moved slower in the NOR test compared to the open field, they still covered higher distances at a reasonable speed, indicating that the reduced distance moved could be due to increased time in exploring the objects. Also, no significant differences in distance moved were found between open field (First test) and Y-Maze (third test), and we found no correlation between the distance moved in the open field and any pellet-reaching measurement, indicating that the animals were not affected by fatigue on the subsequent test".

Conflating "Working Memory" with "Activity/Exploration" in the Y-maze: You interpret the Correct Alternation Ratio (CAR) as an index of "working memory" and link it to pellet-reaching success. However, a high CAR in the Y-maze is also intrinsically linked to a higher number of arm entries ($r=0.96$), which is a direct measure of general locomotor activity and exploratory drive. How can you rule out the possibility that the improved pellet-reaching success is not directly due to superior spatial working memory, but rather a correlate of a more active, trial-and-error prone behavioral phenotype? The neural mechanisms underlying these two interpretations could be different.

We thank the reviewer for raising an interesting point. If the correlation between the Y-maze CAR and pellet-reaching success ratio was linked merely to increased activity in the Y-maze, such as trial-and-error, we would expect those variables to be correlated to the number of attempts, which would be the equivalent trial-and-error in the pellet-reaching test. However, we showed that none of the Y-maze measures is correlated to any of the pellet-reaching measures, except Y-maze CAR and pellet-reaching success ratio. Further, we now added a full predictive analysis controlled by both distance moved and total number of possible alternations, and found Y-maze CAR to be a reliable predictor of pellet-reaching success ratio (Figure 4). We added these points to the discussion section (lines 327-336).

"The findings suggest that the relationship between the Y-maze correct alternation ratio and pellet-reaching success ratio is not simply due to increased activity or trial-and-error behavior in the Y-maze. If this was the case, we would expect a correlation between these variables and the number of attempts in the pellet-reaching task, reflecting a general tendency towards trial-and-error across both tests. However, our results indicate no such correlation exists, implying that the connection between the Y-maze correct alternation ratio and pellet-reaching success is likely driven by other factors, such as specific cognitive processes or neural mechanisms, rather than mere activity levels. Further, we found that Y-maze correct alternation ratio is a reliable predictor of pellet-reaching success ratio, even when accounting for distance moved and total number of alternations. This distinction is important because it highlights that the observed relationship is not a byproduct of general exploratory or motor activity, but may instead reflect more targeted aspects of learning or memory functions that are shared between the tasks."

3. On Data Analysis and Statistics

Correlation vs. Causation/Directionality: You report several significant correlations but the direction of interpretation sometimes requires more nuance. For example, the negative correlation between Vertical Pole time-to-turn and the total number of reaches is interpreted as better motor coordination leading to more attempts. Could the reverse, or a third variable, be plausible? For instance, could a higher general motivation level (interest in sugar) or lower baseline anxiety simultaneously cause faster turning on the pole and more attempts in the reaching task?

While our interpretations are grounded in established experimental design and behavioral literature related to the structural and functional reorganization in the upper layers of the motor cortex (10.1038/ncomms15834), which encode a range of motor parameters such as direction, velocity, position, and muscle activity (10.1523/JNEUROSCI.08-08-02913.1988; 10.1152/jn.1995.73.2.83; 10.1152/jn.00587.2002; 10.1152/jn.1999.82.5.267; 10.1152/jn.01086.2005), after we performed the re-analysis suggested in the Reviewer #2 comment on Line 190, we found the vertical-pole measures to not be correlated with the pellet-reaching measures, and therefore removed all claims based on the correlation between vertical pole time to turn and pellet reaching performance.

Clarity in Statistical Reporting: P-values in Table 1: In the provided table, many p-values are listed as "p=0.0". This is statistically improbable and likely a display artifact. Please verify and report all p-values accurately (e.g., as $p < 0.001$).

We thank the reviewer for noticing the display artifact. The only instances of "p =0.0" in the table are in the diagonal (showing the correlation of each measure with itself), which will always result in a perfect correlation. All significant p-values in this study are already accurately reported in scientific notation (e.g., 1.2e-04 instead of 0.00012). To avoid confusion, we removed the statistics from the diagonal of the table.

Multiple Comparisons: You state that no multiplicity correction was applied due to the exploratory nature of the full correlation matrix, which is acceptable. However, you highlight five specific significant correlations in the results. Did you consider calculating the False Discovery Rate (FDR) to assess the reliability of these "significant" findings within an exploratory framework? This is particularly important given the modest sample size.

We did not apply multiplicity correction because doing so would misalign statistical inference with scientific interpretation (10.1016/j.metip.2023.100120; 10.1016/j.metip.2024.100140). FDR correction (or any other multiple-testing correction) controls only the expected proportion of false positives across a set of tests under an omnibus null hypothesis, not of false positives on individual pairwise testing. In our study, no broad inferences were made (e.g., "all correlations are zero", or "all pre-screening measures correlate with all pellet-reaching outcomes"), therefore applying FDR to individual pairwise correlations would not correct the Type-I error rate and would inadequately inflate the Type II error rate. Particularly in correlation analysis, where a significant correlation does not necessarily mean a meaningful relation (p alone without considering R), incorrectly decreasing the alpha level only increases the distance between the statistical result and its biological meaning. We added a summary of this discussion to the methods section (lines 386-394):

"While multiple comparisons are commonly addressed through multiplicity corrections, these are primarily designed to control false positives under a global null hypothesis, such as "all pre-screening

measures correlate with motor learning outcomes". In exploratory studies where no broad inferences are made across multiple pairwise relationships, applying multiplicity correction to individual correlations do not appropriately adjust for Type I error rates, and instead inflate Type II errors by reducing statistical power, increasing the distance between observed correlations and their biological meaning. Given that our analysis focused on identifying specific, interpretable patterns rather than testing a comprehensive set of interrelationships among behavioral measures, we interpret the reported correlations as exploratory findings grounded in effect size (R) and contextual plausibility, rather than relying solely on p-values or multiplicity-adjusted thresholds."

4. On Sample and Generalizability

Sample Size and Statistical Power: The final sample size for key analyses is 11-12 mice, and only 9 for NOR. While some strong correlations were found (e.g., r=0.79 between Y-maze CAR and success ratio), the small sample may be underpowered to detect moderate or weak correlations and may render the estimates of the found correlations unstable. Did you perform a priori or post hoc power analysis? For the many task pairs where no correlation was found (most of Table 1), is the lack of association genuine, or a potential Type II error due to low power?

Yes, we performed a post-hoc power analysis, and found sufficient power for the main finding that Y-maze and pellet-reaching performances are correlated (r = 0.93, power = 0.997). We also added a comprehensive predictive analysis showing bootstrap distributions and confidence intervals, further confirming that Y-maze performance is a reliable predictor of pellet reaching performance. We agree that lack of correlation could be due to a potential Type II error, which is why we did not draw any conclusions from the negative findings in Table 1, as stated in the discussion lines 358-361.

"While our study provides valuable insights into the relationship between contextual memory and motor learning, it is important to acknowledge that several factors may influence the interpretation of results. One limitation is the relatively small sample size, which although powerful enough to find the correlations shown here, could not be powerful enough to detect more nuanced behavioral correlations."

Single-Sex Limitation: The study uses only female mice, justified by avoiding male aggression and citing literature showing no sex differences in the baseline metrics used. However, in motor learning, especially involving reward and fine motor skills, the estrous cycle can modulate motivation, activity, and learning rate. While the cited literature supports a lack of difference in the individual tasks, the cross-task correlations themselves remain unvalidated across sexes. This limits the generalizability of the conclusions. This limitation should be more prominently discussed.

We acknowledge the reviewer's insightful comment regarding the estrous cycle in our study. We agree that in motor learning tasks, particularly those involving reward, motivation, and fine motor skills, the estrous cycle can influence behavioral outcomes and although some literature shows no influence of the estrous cycle in C57BL/6 female mouse behaviors (10.3389/fnmol.2023.1146109); only in stress (10.1016/j.bbr.2012.11.040), other studies suggests the influence of stress on reward learning is affected by the estrous cycle (10.1038/s41386-025-02170-8) being an important factor to consider. We now discuss these points in the discussion section (lines 363-368).

"Although there is no difference in performance in C57BL/6 female mouse behavior due to the estrous cycle on exploratory measures (10.3389/fnmol.2023.1146109), it could be important to consider the estrous phase in learning tasks since the influence of stress on reward learning is affected by the estrous

cycle (10.1038/s41386-025-02170-8), and estrogen could modulate reward prediction errors and reinforce learning (10.1038/s41593-025-02104-z). Future research should consider the potential impact of estrous cycle phases, thereby improving the generalizability and translational relevance of our results".

5. Other Specific Points

Evidence of "Learning" in the Pellet Reaching Task: The task is described as assessing "fine motor learning," but the results primarily report aggregate measures (total successes, attempts) over three test days. Is there data showing a within-subject improvement in success ratio across the three days? Presenting learning curves (even if non-significant due to few days) would better support that "learning" occurred, not just differences in "performance."

While we agree that learning curves would provide stronger evidence for within-subject improvement in performance, early motor skill acquisition is characterized by high error rates and trial-and-error behavior, which can temporarily suppress success ratios despite underlying learning (10.3791/51238). When analyzing pellet-reaching data across the three test days, we observed a non-significant trend toward increased total successful reaches (ANOVA, $F(2,18) = 2.985$, $p = 0.076$) and increase in total attempts ($F(2,18) = 2.812$, $p = 0.087$). As a result, the overall success ratio remained flat across sessions. We emphasize that a flat success ratio in the early learning stage does not imply absence of learning, rather, it reflects the fact that while animals increase their effort (more attempts), they do not yet achieve a proportional improvement in accuracy during early acquisition phases. Therefore, we chose to report aggregate measures over the three test days, as they provide a more stable and interpretable summary of overall skill acquisition during this early learning stage.

Dissociation of "Turn" vs. "Descent" in the Vertical Pole: The interesting finding that only time-to-turn, not time-to-descend, correlates with pellet reaching is interpreted as the turn requiring finer motor control. Is there any kinematic analysis or behavioral observation to support this mechanistic interpretation? Otherwise, a "fear of heights" could plausibly affect both turning and descending latency.

Thank you for your insightful comment. As explained above, after we performed the re-analysis suggested in the Reviewer #2 comment on Line 190, we found the vertical-pole measures to not be correlated with the pellet-reaching measures, and therefore removed all claims based on the correlation between vertical pole time to turn and pellet reaching performance.

Reviewer #2

I think this exploration of cross -assay performance in mice is interesting, and well-presented but ultimately this study is heavily limited, and requires substantial revision. The sample size is very small, which is understandable, but combined with the analysis methods used reduce confidence in the results. There is a big issue with correlation across independent run sub correlations with different sample sizes. There are also results of a slew of variables that do not directly address the question, think could be removed to focus.

Conceptually as well, this study is unconvincing. I think many of the results could be attributed to behavioural trait linked to speed/ activity rather than memory/ motor skills. Many of the methods used to describe the assays and procedures are solid, but then the measures used are not well justified and do not clearly separate activity from actual cognitive performance.

We appreciate the reviewer's engagement with our work. We respectfully disagree with the characterization of this study as being "heavily limited" or "conceptually unconvincing" due to methodological choices, particularly the inclusion of standard behavioral measures that are widely accepted in neuroscience research. The use of established tasks, such as the Y-maze for spatial working memory (10.1007/978-1-4939-8994-2_10) and the vertical pole task for fine motor coordination (10.1016/j.bbi.2011.06.015; 10.1016/S0165-0270(96)02211-X; 10.17504/protocols.io.5qpvo34k9v4o/v1), is not arbitrary; these protocols have been validated across numerous studies in mice, with clear distinctions between activity levels and specific behavioral outcomes. The correlations we reported are definitely not driven by general locomotion, the distance moved were evaluated and reported for all arenas where it is possible to do so, and were included in the full correlation matrix. Further, we now included a full predictive validity analysis using partial correlations, multiple linear regression, leave-one-out cross-validation (LOOCV), and bootstrap confidence intervals, showing that Y-maze performance is a reliable predictor of pellet-reaching performance, even when accounting for general activity confounders. The claim that our results could be attributed to speed or activity is not supported by our data nor by previous research employing these tasks.

We also emphasize that every variable presented was selected based on its documented relevance to each task, with no addition of unvalidated or speculative measures. We therefore maintain that all presented variables are both methodologically sound and conceptually justified, and we respectfully request the reviewer reconsider their conclusion that these findings lack validity or significance.

Line 174: why 4 days? Is that sufficient for all to achieve habituation, how do we know?

We habituated and trained the mice for four consecutive days, as previously done (10.3389/fncir.2022.842748), which was sufficient since none of the animals displayed signs of anxiety or fear (such as urinating and defecating in the chamber or trying to escape by jumping towards the chamber ceiling). Further, mice performed a reasonable number of attempts, indicating that both habituation and training were effective. We included this information in the methods (lines 178-180).

"The mice were trained and habituated to the arena for four consecutive days, as previously done (10.3389/fncir.2022.842748), which was sufficient since none of the animals exhibited signs of anxiety or fear, such as urinating and defecating in the chamber or attempting to escape by jumping towards the chamber ceiling."

Line 185: a minimum of ? So there was not a standardised rest interval for all individuals? What was the range of variation in rest days?

We thank the reviewer for the comment, since the description is incorrect. We added to the text that the rest interval was one day, due to the food restriction protocol (food restriction at the end of day 0, test on day 1, food restriction at the end of day 2, test on day 3 and so on).

Line 190: including events when they reach ‘for the pellet when no pellet was available’ is not really a failure that can be attributed to fine motor skill. I would argue that the authors re-run the analysis with these ‘failures’ removed.

We agree, and have re-ran all correlation analysis not accounting for prehensions when pellets were not available. All conclusions were maintained, except the correlations between pellet reaching and vertical pole tasks. After re-analysis, we found the vertical-pole measures to not be correlated with the pellet-reaching measures, and therefore removed all claims based on the correlation between vertical pole time to turn and pellet reaching measures.

Line 194: …Anova’s as multiple ones were used. But see below.

See below for the reasoning on using two separate one-way repeated measures ANOVAs to separately evaluate the NOR object familiarity and the distance/speed across different arenas.

Methods: There are two important general things, as this analysis section is both overly complex and also not detailed enough to be reproducible. First, as it was the same individuals, this data would be better analysed in a single model not as separate one-way Anova’s it would allow variation introduced by individual differences to be better accounted for within and between tests, and to better account for the different sample sizes across tests.

Analyzing data from the same individuals in a single multivariate model combining NOR exploration time and total distance moved across arenas is not statistically valid (or possible) under this experimental design. The two behavioral outcomes are derived from distinct tasks with different measurement structures: NOR exploration time involves 18 observations (9 subjects, each with one "familiar" and one "novel" measure), while total distance moved includes 27 observations across three arenas (same 9 subjects evaluated in OF, NOR, and Y-maze). Note that each exploration time measurement occurs in a different context than any distance measurement, and there is no simultaneous recording of both variables for the same subject during the same trial. Without this alignment, combining these outcomes into a multivariate model such as MANOVA (e.g., with a formula "ExplorationTime + DistanceMoved ~ NORStimType * Arena") is not possible; even if the number of observations were artificially adjusted to match, it would introduce statistical bias, distort within-subject variability estimates, and lead to invalid inferences about group differences or individual variation. Therefore, separate one-way repeated measures ANOVAs are appropriate because they allow for clear interpretation of specific effects (such as object familiarity or arena type) and avoid unwarranted assumptions about shared response patterns across unaligned outcomes.

As to the second point, the level of detail varied widely. The text on lines197 – 200 about basic regression is redundant for an academic audience and is extremely basic.

We agreed and removed the redundant information.

But then there is not detail about how the authors '… computed the multiple correlation coefficient based on their pairwise correlation coefficients, representing the shared linear variance.'

Although the code used to compute the triple correlation was already available, after re-running all correlation analysis as suggested in the Reviewer #2 comment on Line 190, we found the vertical-pole measures to not be correlated with the pellet-reaching measures, and therefore removed all claims based on the correlation between vertical pole time to turn and pellet reaching performance.

Line 202: why use a triple correlation coefficient computed from the pre-existing pairwise correlation coefficients rather than from the raw data ? It is not standard practice, and readers need to know what formula were used. Far more importantly, Deriving these pairwise correlations assumes those correlations were computed on the same sample sizes with no missing data, and any pairwise correlation using different subsets of individuals as is the case with this study the result would be hard to interpret at best and potentially invalid.

Although we respectfully disagree that using pairwise correlations to compute a multiple correlation coefficient is non-standard, since this approach is not only valid, but defined in standard multivariate statistics literature (e.g., 10.1533/9780857097200.631, Problem 7.4, Equation 7.23; the multiple correlation coefficient between three outcomes based on shared predictors is derived from their pairwise correlations); and the reviewer's explicit statement that our study uses "different subsets of individuals" is misleading, since the difference between the animals used for the pairwise correlations used in the triple correlation is only one mouse (included in the vertical pole time to turn vs. Y-maze correct alternation ratio correlation, but not present in the other two pairwise correlations); after re-running all correlation analysis as suggested in the Reviewer #2 comment on Line 190, we found the vertical-pole measures to not be correlated with the pellet-reaching measures, and therefore removed all claims based on the correlation between vertical pole time to turn and pellet reaching performance.

Line 214: So no videos were manually scored? If not how do you (and thus we) know that Ethovision was working correctly and effectively distinguishing between different behaviours? I would ask the authors what aspect of research they actually wanted to engage in.

For behavioral data tracking and extraction in the open field, novel object recognition, and Y-maze tasks, we used Ethovision, a well-established, peer-reviewed software (10.3758/BF03195394), specifically designed for automated behavioral analysis. This tool has been extensively validated across multiple laboratories, including our own (10.1016/j.neuron.2018.06.022). We chose to use Ethovision instead of manual scoring for these tasks, which is prone to observer bias, inter-rater variability, time-intensive labor, and inconsistent temporal resolution. Furthermore, all analysis parameters for the experiments using Ethovision are clearly stated on each task's method section. We would answer the proposed question by saying that our work focuses on behavioral correlations between possible pre-screening tasks and a motor learning task, not on re-validating analytical tools that are already extensively validated. We believe that questioning whether our research engaged with software validation misrepresents both the scope and purpose of this study.

Line 240: This is not an outlier, unless you measurements were off for this one individual. It is very likely that this reflects actual relevant among-individual variation within the group. Treating it and or naming it an outlier is terminologically incorrect and conceptually odd. I am glad the measurements from this individual (I assume – the wording is slightly ambiguous) was kept in the analysis as the sample size is already very low for this study, but the authors must be careful with what they term a statistical outlier. Especially when comparing a data point to a group average – which is not a method that can be used to determine a statistical outlier.

A statistical outlier is a data point that differs from the other points in the same set according to objective measures. The distance moved for the mouse mentioned above, for example in the open field, is 2.17 standard deviations above the mean and 3.92 × IQR above the third quartile of the dataset, both of which meet established, widely used criteria for statistical outliers (Tukey, 1977, ISBN 0-201-07616-0). We have now explicitly stated these values in the Results section (lines 262-264) to ensure full transparency about how this point was identified as a statistical outlier under objective criteria. Regarding the inclusion of this mouse's data, we respectfully disagree that the wording is ambiguous, since we already clearly stated in the previous version lines 242-243: "Since the mouse identified as a statistical outlier did not display immobility during the tasks, no animal was excluded from further analysis." (current version lines 264-266).

Line 253: 'escape responses – what are these ? Also this makes sense from the perspective of 'declarative' memory - more successful maze entries = good memory and less time spent exploring novel object. But this depends on a more refined measure of novel object exploration time (i.e. relative duration of time spent exploring a novel object does not say much – if some individuals just take longer to approach. Also, how much time do we expect a mouse to take to satisfy their curiosity about a novel object. Were the ones that spent longer in the investigation area actually inefficient? I think interpreting this test as measured is prone to ambiguity.

"Escape responses" refer to repeated or persistent entries into the Y-maze arms without a clear pattern, indicating a tendency for mice to engage in exploratory behavior rather than the correct alternation of arm entries. While we observed a negative correlation between the number of arm entries and the time spent exploring the novel object, this does not imply inefficiency. Instead, it suggests mice with more escape behavior may prioritize navigation over sensory engagement. We respectfully disagree that the relative exploration time is not a reliable measure due to differences in approach time, since only the exploration time is considered. The ratio presented here, as described in the NOR methods section (lines 136-138), is the time spent investigating the novel object relative to the total object investigation time (recognition index) and it is the main score for retention (10.1007/s10339-011-0430-z). This metric better reflects mice ability to recognize the new object than arbitrary time limits, and our interpretation remains consistent with established norms in NOR studies (e.g., 10.1016/j.beproc.2009.10.003, 10.1016/S0166-4328(01)00444-2, 10.1101/lm.1035508, 10.1038/npp.2010.67).

Line 254: higher *number of* arm entries.

Thanks, corrected.

I note that the data are to be provided upon acceptance.

We appreciate it, and kindly ask to change the answer to the question “3. Have the authors made all data underlying the findings in their manuscript fully available?” to “Yes”, since all data points for each mouse across all tasks are already present in Figure 1, and additional files for easier data access will be provided upon acceptance.
