# Supplementary material for: Y-maze performance predicts refined motor learning in mice
Source: PLoS One. 2026 Jul 1;21(7):e0352676. doi: 10.1371/journal.pone.0352676 (PMC13322532; doi:10.1371/journal.pone.0352676)
Supplement: Peer review history

# Peer review: Decision Letter 0

**DOI:** 10.1371/journal.pone.0352676.r001

**Authors:** Stephen Ginsberg

20 Mar 2026

PONE-D-26-07602

Y-maze and Vertical Pole Performance Correlate with Fine Motor Learning in Mice

PLOS One

Dear Dr. Ciralli,

Thank you for submitting your manuscript to PLOS ONE. After careful consideration, we feel that it has merit but does not fully meet PLOS ONE’s publication criteria as it currently stands. Therefore, we invite you to submit a revised version of the manuscript that addresses the points raised during the review process.

Please submit your revised manuscript by May 02 2026 11:59PM. If you will need more time than this to complete your revisions, please reply to this message or contact the journal office at plosone@plos.org. When you're ready to submit your revision, log on to https://www.editorialmanager.com/pone/ and select the 'Submissions Needing Revision' folder to locate your manuscript file.

Please include the following items when submitting your revised manuscript:

- A letter that responds to each point raised by the academic editor and reviewer(s). You should upload this letter as a separate file labeled 'Response to Reviewers'.
- A marked-up copy of your manuscript that highlights changes made to the original version. You should upload this as a separate file labeled 'Revised Manuscript with Track Changes'.
- An unmarked version of your revised paper without tracked changes. You should upload this as a separate file labeled 'Manuscript'.

If you would like to make changes to your financial disclosure, please include your updated statement in your cover letter. Guidelines for resubmitting your figure files are available below the reviewer comments at the end of this letter.

If applicable, we recommend that you deposit your laboratory protocols in protocols.io to enhance the reproducibility of your results. Protocols.io assigns your protocol its own identifier (DOI) so that it can be cited independently in the future. For instructions see: https://journals.plos.org/plosone/s/submission-guidelines#loc-laboratory-protocols. Additionally, PLOS ONE offers an option for publishing peer-reviewed Lab Protocol articles, which describe protocols hosted on protocols.io. Read more information on sharing protocols at https://plos.org/protocols?utm_medium=editorial-email&utm_source=authorletters&utm_campaign=protocols.

We look forward to receiving your revised manuscript.

Kind regards,

Stephen D. Ginsberg, Ph.D.

Section Editor

PLOS One

Journal Requirements:

When submitting your revision, we need you to address these additional requirements.

1.Please ensure that your manuscript meets PLOS ONE's style requirements, including those for file naming. The PLOS ONE style templates can be found at

https://journals.plos.org/plosone/s/file?id=wjVg/PLOSOne_formatting_sample_main_body.pdf and

https://journals.plos.org/plosone/s/file?id=ba62/PLOSOne_formatting_sample_title_authors_affiliations.pdf

2. To comply with PLOS One submissions requirements, in your Methods section, please provide additional information regarding the experiments involving animals and ensure you have included details on (1) methods of sacrifice, (2) methods of anesthesia and/or analgesia, and (3) efforts to alleviate suffering.

3. We note that the grant information you provided in the ‘Funding Information’ and ‘Financial Disclosure’ sections do not match.

When you resubmit, please ensure that you provide the correct grant numbers for the awards you received for your study in the ‘Funding Information’ section.

4. Thank you for stating the following financial disclosure:

“We thank the Swedish Research Council (2018–02750, 2022-01245; www.vr.se), the Swedish Brain Foundation (FO2020-0228, FO2022-0018; http://hjarnfonden.se), U-Share (2021.1.1.1-4394; www.u-share.se), and Olle Engkvist Byggmästare Foundation (220-0254; https://engkviststiftelserna.se).”

Please state what role the funders took in the study.  If the funders had no role, please state: "The funders had no role in study design, data collection and analysis, decision to publish, or preparation of the manuscript."

If this statement is not correct you must amend it as needed.

Please include this amended Role of Funder statement in your cover letter; we will change the online submission form on your behalf.

5. When completing the data availability statement of the submission form, you indicated that you will make your data available on acceptance. We strongly recommend all authors decide on a data sharing plan before acceptance, as the process can be lengthy and hold up publication timelines. Please note that, though access restrictions are acceptable now, your entire data will need to be made freely accessible if your manuscript is accepted for publication. This policy applies to all data except where public deposition would breach compliance with the protocol approved by your research ethics board. If you are unable to adhere to our open data policy, please kindly revise your statement to explain your reasoning and we will seek the editor's input on an exemption. Please be assured that, once you have provided your new statement, the assessment of your exemption will not hold up the peer review process.

6. Please note that funding information should not appear in any section or other areas of your manuscript. We will only publish funding information present in the Funding Statement section of the online submission form. Please remove any funding-related text from the manuscript.

7. Please amend your list of authors on the manuscript to ensure that each author is linked to an affiliation. Authors’ affiliations should reflect the institution where the work was done (if authors moved subsequently, you can also list the new affiliation stating “current affiliation:….” as necessary).

8. Please include captions for your Supporting Information files at the end of your manuscript, and update any in-text citations to match accordingly. Please see our Supporting Information guidelines for more information: http://journals.plos.org/plosone/s/supporting-information.

9. If the reviewer comments include a recommendation to cite specific previously published works, please review and evaluate these publications to determine whether they are relevant and should be cited. There is no requirement to cite these works unless the editor has indicated otherwise.

[Note: HTML markup is below. Please do not edit.]

Reviewers' comments:

Reviewer's Responses to Questions

**Comments to the Author**

1. Is the manuscript technically sound, and do the data support the conclusions?

The manuscript must describe a technically sound piece of scientific research with data that supports the conclusions. Experiments must have been conducted rigorously, with appropriate controls, replication, and sample sizes. The conclusions must be drawn appropriately based on the data presented.

Reviewer #1: Yes

Reviewer #2: No

**********

2. Has the statistical analysis been performed appropriately and rigorously?

Reviewer #1: N/A

Reviewer #2: No

**********

3. Have the authors made all data underlying the findings in their manuscript fully available?

The PLOS Data policy requires authors to make all data underlying the findings described in their manuscript fully available without restriction, with rare exception (please refer to the Data Availability Statement in the manuscript PDF file). The data should be provided as part of the manuscript or its supporting information, or deposited to a public repository. For example, in addition to summary statistics, the data points behind means, medians and variance measures should be available. If there are restrictions on publicly sharing data—e.g. participant privacy or use of data from a third party—those must be specified.

Reviewer #1: Yes

Reviewer #2: No

**********

4. Is the manuscript presented in an intelligible fashion and written in standard English?

PLOS ONE does not copyedit accepted manuscripts, so the language in submitted articles must be clear, correct, and unambiguous. Any typographical or grammatical errors should be corrected at revision, so please note any specific errors here.

Reviewer #1: Yes

Reviewer #2: Yes

**********

5. Review Comments to the Author

Please use the space provided to explain your answers to the questions above. You may also include additional comments for the author, including concerns about dual publication, research ethics, or publication ethics. (Please upload your review as an attachment if it exceeds 20,000 characters)

Reviewer #1: To strengthen the rigor and impact of your findings, I have several critical points that require clarification and further consideration.

1. On the Core Conclusion and the Claim of "Prediction"

The central conclusion posits that the Y-maze and Vertical Pole tasks are "suitable predictors" of pellet-reaching performance. However, the study design is inherently cross-sectional and correlational, not longitudinal or predictive. All tasks were assessed within a similar timeframe. Can you provide stronger justification or refine the language to clarify how a correlation measured concurrently translates into a reliable predictionof future performance on a distinct, more complex task? Is the implied predictive power based on shared neural substrates (e.g., hippocampal-striatal circuits) or a more general "learning readiness" trait? The term "predictor" may be too strong without evidence from a true pre-screening followed by a later assessment of motor learning.

2. On Experimental Design and Confounding Factors

Fixed Order of Testing: The Methods indicate that all behavioral assays (Open Field, NOR, Y-maze, Vertical Pole) were conducted prior to the Pellet Reaching task. This fixed order is a significant confound. Could the testing sequence itself introduce practice effects, fatigue, or cumulative stress that systematically influences performance in the final, most complex task? For instance, are mice that are more active/less anxious in the initial tasks also the ones that show higher engagement in the final motor learning task? While you briefly acknowledge this in the discussion (lines 378-386), a deeper analysis is needed to disentangle whether the observed correlations reflect intrinsic ability links or are artifacts of the testing schedule.

Conflating "Working Memory" with "Activity/Exploration" in the Y-maze: You interpret the Correct Alternation Ratio (CAR) as an index of "working memory" and link it to pellet-reaching success. However, a high CAR in the Y-maze is also intrinsically linked to a higher number of arm entries (r=0.96), which is a direct measure of general locomotor activity and exploratory drive. How can you rule out the possibility that the improved pellet-reaching success is not directly due to superior spatial working memory, but rather a correlate of a more active, trial-and-error prone behavioral phenotype? The neural mechanisms underlying these two interpretations could be different.

3. On Data Analysis and Statistics

Correlation vs. Causation/Directionality: You report several significant correlations but the direction of interpretation sometimes requires more nuance. For example, the negative correlation between Vertical Pole time-to-turnand the total number of reachesis interpreted as better motor coordination leading to more attempts. Could the reverse, or a third variable, be plausible? For instance, could a higher general motivation level (interest in sugar) or lower baseline anxiety simultaneously cause faster turning on the pole andmore attempts in the reaching task?

Clarity in Statistical Reporting:

P-values in Table 1: In the provided table, many p-values are listed as "p=0.0". This is statistically improbable and likely a display artifact. Please verify and report all p-values accurately (e.g., as p < 0.001).

Multiple Comparisons: You state that no multiplicity correction was applied due to the exploratory nature of the full correlation matrix, which is acceptable. However, you highlight five specific significant correlations in the results. Did you consider calculating the False Discovery Rate (FDR) to assess the reliability of these "significant" findings within an exploratory framework? This is particularly important given the modest sample size.

4. On Sample and Generalizability

Sample Size and Statistical Power: The final sample size for key analyses is 11-12 mice, and only 9 for NOR. While some strong correlations were found (e.g., r=0.79 between Y-maze CAR and success ratio), the small sample may be underpowered to detect moderate or weak correlations and may render the estimates of the found correlations unstable. Did you perform a priori or post hoc power analysis? For the many task pairs where no correlation was found (most of Table 1), is the lack of association genuine, or a potential Type II error due to low power?

Single-Sex Limitation: The study uses only female mice, justified by avoiding male aggression and citing literature showing no sex differences in the baseline metrics used. However, in motor learning, especially involving reward and fine motor skills, the estrous cycle can modulate motivation, activity, and learning rate. While the cited literature supports a lack of difference in the individual tasks, the cross-task correlations themselves remain unvalidated across sexes. This limits the generalizability of the conclusions. This limitation should be more prominently discussed.

5. Other Specific Points

Evidence of "Learning" in the Pellet Reaching Task: The task is described as assessing "fine motor learning," but the results primarily report aggregate measures (total successes, attempts) over three test days. Is there data showing a within-subject improvement in success ratio across the three days? Presenting learning curves (even if non-significant due to few days) would better support that "learning" occurred, not just differences in "performance."

Dissociation of "Turn" vs. "Descent" in the Vertical Pole: The interesting finding that only time-to-turn, not time-to-descend, correlates with pellet reaching is interpreted as the turn requiring finer motor control. Is there any kinematic analysis or behavioral observation to support this mechanistic interpretation? Otherwise, a "fear of heights" could plausibly affect both turning and descending latency.

Reviewer #2: I think this exploration of cross -assay performance in mice is interesting, and well-presented but ultimately this study is heavily limited, and requires substantial revision. The sample size is very small, which is understandable, but combined with the analysis methods used reduce confidence in the results. There is a big issue with correlation across independent run sub correlations with different sample sizes. There are also results of a slew of variables that do not directly address the question, think could be removed to focus.

Conceptually as well, this study is unconvincing. I think many of the results could be attributed to behavioural trait linked to speed/ activity rather than memory/ motor skills. Many of the methods used to describe the assays and procedures are solid, but then the measures used are not well justified and do not clearly separate activity from actual cognitive performance.

Line 174: why 4 days? Is that sufficient for all to achieve habituation, how do we know?

Line 185: a minimum of ? So there was not a standardised rest interval for all individuals? What was the range of variation in rest days?

Line 190: including events when they reach ‘for the pellet when no pellet was available’ is not really a failure that can be attributed to fine motor skill. I would argue that the authors re-run the analysis with these ‘failures’ removed.

Line 194: …Anova’s as multiple ones were used. But see below.

Methods: There are two important general things, as this analysis section is both overly complex and also not detailed enough to be reproducible. First, as it was the same individuals, this data would be better analysed in a single model not as separate one-way Anova’s it would allow variation introduced by individual differences to be better accounted for within and between tests, and to better account for the different sample sizes across tests. As to the second point, the level of detail varied widely. The text on lines197 – 200 about basic regression is redundant for an academic audience and is extremely basic. But then there is not detail about how the authors ‘… computed the multiple correlation coefficient based on their pairwise correlation coefficients, representing the shared linear variance.’

Line 202: why use a triple correlation coefficient computed from the pre-existing pairwise correlation coefficients rather than from the raw data ? It is not standard practice, and readers need to know what formula were used. Far more importantly, Deriving these pairwise correlations assumes those correlations were computed on the same sample sizes with no missing data, and any pairwise correlation using different subsets of individuals as is the case with this study the result would be hard to interpret at best and potentially invalid.

Line 214: So no videos were manually scored? If not how do you ( and thus we) know that Ethovision was working correctly and effectively distinguishing between different behaviours? I would ask the authors what aspect of research they actually wanted to engage in.

Line 240: This is not an outlier, unless you measurements were off for this one individual. It is very likely that this reflects actual relevant among-individual variation within the group. Treating it and or naming it an outlier is terminologically incorrect and conceptually odd. I am glad the measurements from this individual (I assume – the wording is slightly ambiguous) was kept in the analysis as the sample size is already very low for this study, but the authors must be careful with what they term a statistical outlier. Especially when comparing a data point to a group average – which is not a method that can be used to determine a statistical outlier.

Line 253: ‘escape responses – what are these ? Also this this makes sense from the perspective of ‘declarative’ memory - more successful maze entries = good memory and less time spent exploring novel object. But this depends on a more refined measure of novel object exploration time (i.e. relative duration of time spent exploring a novel object does not say much – if some individuals just take longer to approach. Also, how much time do we expect a mouse to take to satisfy their curiosity about a novel object. Were the ones that spent longer in the investigation area actually inefficient? I think interpreting this test as measured is prone to ambiguity.

Line 254: higher *number of* arm entries.

I note that the data are to be provided upon acceptance.

**********

6. PLOS authors have the option to publish the peer review history of their article (what does this mean?). If published, this will include your full peer review and any attached files.

If you choose “no”, your identity will remain anonymous but your review may still be made public.

**Do you want your identity to be public for this peer review?**  For information about this choice, including consent withdrawal, please see our Privacy Policy.

Reviewer #1: **Yes:** Yixuan Ku

Reviewer #2: No

**********

[NOTE: If reviewer comments were submitted as an attachment file, they will be attached to this email and accessible via the submission site. Please log into your account, locate the manuscript record, and check for the action link "View Attachments". If this link does not appear, there are no attachment files.]

To ensure your figures meet our technical requirements, please review our figure guidelines: https://journals.plos.org/plosone/s/figures

You may also use PLOS’s free figure tool, NAAS, to help you prepare publication quality figures: https://journals.plos.org/plosone/s/figures#loc-tools-for-figure-preparation.

NAAS will assess whether your figures meet our technical requirements by comparing each figure against our figure specifications.

# Author comment: Author response to Decision Letter 1

**DOI:** 10.1371/journal.pone.0352676.r002

1 Jun 2026

A letter that responds to each point raised by the academic editor and reviewers was uploaded as a separate file labeled 'Response to Reviewers'.

Attachment Submitted filename: 20260525-CiralliEtAl-Reviews_PLOSOne.pdf

# Peer review: Decision Letter 1

**DOI:** 10.1371/journal.pone.0352676.r003

**Authors:** Stephen Ginsberg, Stephen Ginsberg

15 Jun 2026

Y-maze performance predicts refined motor learning in mice

PONE-D-26-07602R1

Dear Dr. Ciralli,

We’re pleased to inform you that your manuscript has been judged scientifically suitable for publication and will be formally accepted for publication once it meets all outstanding technical requirements.

Within one week, you’ll receive an e-mail detailing the required amendments. When these have been addressed, you’ll receive a formal acceptance letter and your manuscript will be scheduled for publication.

An invoice will be generated when your article is formally accepted. Please note, if your institution has a publishing partnership with PLOS and your article meets the relevant criteria, all or part of your publication costs will be covered. Please make sure your user information is up-to-date by logging into Editorial Manager at Editorial Manager® and clicking the ‘Update My Information' link at the top of the page. For questions related to billing, please contact billing support.

If your institution or institutions have a press office, please notify them about your upcoming paper to help maximize its impact. If they’ll be preparing press materials, please inform our press team as soon as possible -- no later than 48 hours after receiving the formal acceptance. Your manuscript will remain under strict press embargo until 2 pm Eastern Time on the date of publication. For more information, please contact onepress@plos.org.

Kind regards,

Stephen D. Ginsberg, Ph.D.

Section Editor

PLOS One

**Comments to the Author**

1. If the authors have adequately addressed your comments raised in a previous round of review and you feel that this manuscript is now acceptable for publication, you may indicate that here to bypass the “Comments to the Author” section, enter your conflict of interest statement in the “Confidential to Editor” section, and submit your "Accept" recommendation.

Reviewer #2: All comments have been addressed

**********

2. Is the manuscript technically sound, and do the data support the conclusions?

The manuscript must describe a technically sound piece of scientific research with data that supports the conclusions. Experiments must have been conducted rigorously, with appropriate controls, replication, and sample sizes. The conclusions must be drawn appropriately based on the data presented.

Reviewer #2: Yes

**********

3. Has the statistical analysis been performed appropriately and rigorously?

Reviewer #2: Yes

**********

4. Have the authors made all data underlying the findings in their manuscript fully available?

The PLOS Data policy requires authors to make all data underlying the findings described in their manuscript fully available without restriction, with rare exception (please refer to the Data Availability Statement in the manuscript PDF file). The data should be provided as part of the manuscript or its supporting information, or deposited to a public repository. For example, in addition to summary statistics, the data points behind means, medians and variance measures should be available. If there are restrictions on publicly sharing data—e.g. participant privacy or use of data from a third party—those must be specified.

Reviewer #2: Yes

**********

5. Is the manuscript presented in an intelligible fashion and written in standard English?

PLOS ONE does not copyedit accepted manuscripts, so the language in submitted articles must be clear, correct, and unambiguous. Any typographical or grammatical errors should be corrected at revision, so please note any specific errors here.

Reviewer #2: Yes

**********

6. Review Comments to the Author

Please use the space provided to explain your answers to the questions above. You may also include additional comments for the author, including concerns about dual publication, research ethics, or publication ethics. (Please upload your review as an attachment if it exceeds 20,000 characters)

Reviewer #2: I think the authors have addressed all the issues raised, and the re framing around the new results is good. Also, I note that two of my earlier comments were not worded very well at all, and might have come across as more critical than intended, my apologies to the authors.

**********

7. PLOS authors have the option to publish the peer review history of their article (what does this mean?). If published, this will include your full peer review and any attached files.

If you choose “no”, your identity will remain anonymous but your review may still be made public.

**Do you want your identity to be public for this peer review?**  For information about this choice, including consent withdrawal, please see our Privacy Policy.

Reviewer #2: No

# Editor report: Acceptance letter

**DOI:** 10.1371/journal.pone.0352676.r004

**Authors:** Stephen Ginsberg, Stephen Ginsberg

PONE-D-26-07602R1

PLOS One

Dear Dr. Ciralli,

I'm pleased to inform you that your manuscript has been deemed suitable for publication in PLOS One. Congratulations! Your manuscript is now being handed over to our production team.

At this stage, our production department will prepare your paper for publication. This includes ensuring the following:

* All references, tables, and figures are properly cited

* All relevant supporting information is included in the manuscript submission,

* There are no issues that prevent the paper from being properly typeset

You will receive further instructions from the production team, including instructions on how to review your proof when it is ready. Please keep in mind that we are working through a large volume of accepted articles, so please give us a few days to review your paper and let you know the next and final steps.

Lastly, if your institution or institutions have a press office, please let them know about your upcoming paper now to help maximize its impact. If they'll be preparing press materials, please inform our press team within the next 48 hours. Your manuscript will remain under strict press embargo until 2 pm Eastern Time on the date of publication. For more information, please contact onepress@plos.org.

You will receive an invoice from PLOS for your publication fee after your manuscript has reached the completed accept phase. If you receive an email requesting payment before acceptance or for any other service, this may be a phishing scheme. Learn how to identify phishing emails and protect your accounts at https://explore.plos.org/phishing.

If we can help with anything else, please email us at customercare@plos.org.

Thank you for submitting your work to PLOS ONE and supporting open access.

Kind regards,

PLOS ONE Editorial Office Staff

on behalf of

Dr. Stephen D. Ginsberg

Section Editor

PLOS One
